# Comparison of Two Real-Time PCR Assays Targeting Ribosomal Sequences for the Identification of *Cystoisospora belli* in Human Stool Samples

**DOI:** 10.3390/pathogens10081053

**Published:** 2021-08-19

**Authors:** Martin Blohm, Andreas Hahn, Ralf Matthias Hagen, Kirsten Alexandra Eberhardt, Holger Rohde, Gérard Leboulle, Torsten Feldt, Fred Stephen Sarfo, Veronica Di Cristanziano, Hagen Frickmann, Ulrike Loderstädt

**Affiliations:** 1 Department of Microbiology and Hospital Hygiene, Bundeswehr Hospital Hamburg, 20359 Hamburg, Germany; martin2blohm@bundeswehr.org (M.B.); frickmann@bnitm.de (H.F.); 2 Institute of Medical Microbiology, Virology and Hygiene, University Medicine Rostock, 18057 Rostock, Germany; Hahn.andreas@me.com; 3 Department of Microbiology and Hospital Hygiene, Bundeswehr Central Hospital Koblenz, 56070 Koblenz, Germany; ralfmatthiashagen@bundeswehr.org; 4 Institute of Transfusion Medicine, University Medical Center Hamburg-Eppendorf, 20251 Hamburg, Germany; ki.eberhardt@uke.de; 5 Department of Tropical Medicine, Bernhard Nocht Institute for Tropical Medicine, 20359 Hamburg, Germany; 6 Institute of Medical Microbiology, Virology and Hygiene, University Medical Center Hamburg-Eppendorf (UKE), 20251 Hamburg, Germany; rohde@uke.de; 7 Tib MolBiol, Eresburgstraße 22-23, 12103 Berlin, Germany; gleboulle@tib-molbiol.de; 8 Department of Gastroenterology, Hepatology and Infectious Diseases, University Medical Center Düsseldorf, 40225 Düsseldorf, Germany; Torsten.Feldt@med.uni-duesseldorf.de; 9 Department of Medicine, Kwame Nkrumah University of Science and Technology, Kumasi 00233, Ghana; stephensarfo78@gmail.com; 10 Institute of Virology, Faculty of Medicine and University Hospital of Cologne, University of Cologne, 50935 Cologne, Germany; veronica.di-cristanziano@uk-koeln.de; 11 Department of Hospital Hygiene & Infectious Diseases, University Medicine Göttingen, 37075 Göttingen, Germany

**Keywords:** *Cystoisospora belli*, diagnosis, stool, real-time PCR, test comparison, immunosuppression, HIV, AIDS, soldier, deployment, parasite

## Abstract

*Cystoisospora (C.) belli* is a coccidian parasite associated with acute or chronic gastroenteritis in immunocompromised patients. Dissatisfactory sensitivity of microscopy as the diagnostic standard approach has been described. Here, we comparatively evaluated two real-time PCRs targeting ribosomal RNA gene sequences of *C. belli* in stool in a test comparison without a reference standard applying latent class analysis. Therefore, 1000 stool samples from Ghanaian HIV (human immunodeficiency virus) patients (n = 905) as well as military returnees from the tropics (n = 95) were assessed by both assays in parallel. After the exclusion of 33 samples showing PCR inhibition, 29 and 33 positive results were recorded with the 5.8S rRNA gene/ITS-2 sequence PCR and the ITS-2 sequence PCR, respectively, resulting in an accuracy-adjusted prevalence of 3.2%. Nearly perfect agreement between both assays was indicated by Fleiss’ kappa of 0.933 with sensitivity and specificity of 92.8% and 100% as well as 100% and 99.8% for the 5.8S rRNA gene/ITS-2 sequence PCR and the ITS-2 sequence PCR, respectively. Both assays proved to be suitable for the diagnosis of *C. belli* in human stool samples with slightly better sensitivity of the ITS-2 sequence assay, while the 5.8S rRNA gene/ITS-2 sequence PCR may be considered for confirmatory testing.

## 1. Introduction

*Cystoisospora (C.) belli* is an apicomplexan coccidian protozoan parasite causing gastroenteritis in severely immunocompromised patients, e.g., patients with acquired immunodeficiency syndrome (AIDS) as well as patients undergoing immunosuppressive therapy or chemotherapy of tumors [1,2]. In particular, the association of *C. belli*-induced gastroenteritis with AIDS has repeatedly been described from various international study sites [3,4,5,6,7,8,9,10,11,12], while the parasite is rarely isolated from immunocompetent patients with chronic diarrhea [13]. Transmission occurs primarily in the subtropics and tropics via the fecal–oral route due to food or water contaminated with *C. belli* cysts [1]. In local food markets, cockroaches have been identified as likely mechanical vectors of *C. belli* transmission [14]. Further, *Cystoisospora* spp. have been detected in primates in Ghana, but their role in transmission remains uncertain [15]. After infection, the parasites can be found in the epithelium of the intestines, the bile duct and the gallbladder [1,16,17]. In particular for the latter location, however, histological misidentifications have been reported as well [18,19]. In case of systemic dissemination of the parasite in severely immunocompromised patients, *C. belli* can also be found in extra-intestinal locations such as the lamina propria of the small and large intestine, lymphatic nodes, the spleen, the liver and even in blood smears [1,20]. Persisting disease and even relapses of *C. belli*-associated disease, also called cystoisosporiasis, are common in immunocompromised patients [1]. Medical therapy is usually based on cotrimoxazole for 7 to 10 days [1,17].

Although microscopy, usually following acid-fast staining or using the autofluorescing properties of *C. belli*, is the standard diagnostic approach for the detection of *C. belli* in the patients’ stool samples [16,21,22], it has early been argued that more sensitive molecular diagnostic assays may facilitate both diagnostic detection of the parasite and insights into the pathogen’s transmission and mode of persistence [21,23,24]. Molecular diagnostic approaches for the identification and typing of *C. belli* were frequently focused on ribosomal DNA sequences [25]. So, restriction fragment polymorphisms within the small subunit ribosomal RNA (ssu rRNA) gene have been applied for the differentiation between various genotypes of *C. belli* [24]. Restriction length fragment polymorphisms of the internal transcribed spacer 1 (ITS-1) sequence have been used to demonstrate phylogenetic relationship of *Cystoisospora* spp. with cyst-forming coccidia of the family *Sarcocystidae* [26]. Of note, *Sarcocystis* spp. are observed considerably less frequently than other coccidian parasites in stool samples even from immunodeficient human patients with chronic gastroenteritis [27].

Good agreement between traditional microscopic assessment and the molecular diagnosis of *C. belli* has been reported [28]. Different molecular tools have been introduced for the diagnostic detection of *C. belli* in diagnostic specimens [29], comprising extended range PCR screening [30], traditional block-cycler PCR [31], melting-curve-based real-time PCR [32], as well as in-house [28,33] and commercial [34] probe-based real-time PCR assays. One of those in-house probe-based real-time PCR assays [33] has already been successfully applied in epidemiological screenings in tropical high-endemicity settings [35]. Of note, diagnostic sensitivity of molecular assays for *C. belli* was reported to be better with semi-automated nucleic acid extraction applying an EZ1 automate (Qiagen, Hilden, Germany) compared to traditional column-based nucleic acid extraction applying the QIAamp DNA stool mini kit (Qiagen Hilden, Germany) [36].

The two in-house real-time PCR assays for *C. belli* described in recent literature target either a 213-base-pair region comprising *C. belli*’s 5.8S rRNA gene and ITS-2 sequence [28] or an 89-base-pair region within *C. belli*’s ITS-2 sequence [33]. For the 5.8S rRNA gene/ITS-2 protocol, a previous assessment had suggested sensitivity and specificity of 93% each [28], while these values were 100% for the ITS-2-protocol [33] as assessed compared to microscopy with limited numbers of stool samples.

The aim of the study presented here was the comparative evaluation of both described in-house real-time PCR assays in a test comparison without a reference standard.

## 2. Results

### 2.1. Sensitivity and Specificity of the Real-Time PCR Assays as well as Accuracy-Adjusted Prevalence Calculated Applying Latent Class Analysis and Agreement Kappa between the Assays

From 1000 assessed samples, 33 (3.3%) were excluded from the calculations due to recorded sample inhibition. From the remaining 967 samples, the 5.8S rRNA gene/ITS-2 sequence PCR detected *C. belli* in 29 samples (3.0%), the ITS-2 sequence PCR in 33 samples (3.41%). Applying latent class analysis [37,38], sensitivity and specificity of 5.8S rRNA gene/ITS-2 sequence PCR and the ITS-2 sequence PCR were 92.8% and 100.0%, respectively, as well as 100.0% and 99.8%, respectively. However, 95%-confidence intervals comprising the complete percentage spectrum resulted from the low absolute numbers of positive test results. The recorded agreement between the two real-time PCR assays was almost perfect according to the definitions of Landis and Koch [39]. Accuracy-adjusted prevalence of *C. belli* as calculated applying LCA was 3.2% for the study population (Table 1). Thereby, only 1 sample positive by both PCR assays and 2 inhibited samples were recorded among the 95 stool samples obtained from the assessed returned soldiers from deployments in the Democratic Republic of the Congo and Gabon, while all other positive PCR results as well as the remaining 31 inhibited samples were detected within the group of the 905 Ghanaian HIV patients.

### 2.2. Comparison of the Recorded Cycle Threshold (Ct) Values

When comparing the recorded cycle threshold (Ct) values of both *C. belli*-specific real-time PCR assays, both the means and the medians of the Ct values as recorded with both assays were very similar with differences of less than 1 Ct step. The same applied for the recorded standard deviations (Table 2). When focusing on the Ct values in the ITS-2 sequence PCR of the 4 samples that went undetected in the 5.8S rRNA gene/ITS-2 sequence PCR, the recorded values ranged between 33.96 and 35.50 and thus above the recorded Ct mean and median values. However, they were still within the range of the standard deviations and lower than the highest Ct values recorded by the ITS-2 sequence PCR (Table 2).

## 3. Discussion

The study was performed to comparatively assess the diagnostic accuracy of two previously described real-time PCR assays targeting ribosomal sequences of *C. belli* [28,33] in a test comparison without a reference standard [37,38]. To ensure increased pretest-probability, residual DNA eluates from stool samples of HIV patients from tropical Ghana [40,41,42] and of soldiers after tropical deployment in the Democratic Republic of the Congo and Gabon [43] were chosen for the assessments, so *C. belli* infections could be considered as likely [1,2,3,4,5,6,7,8,9,10,11,12]. In people living with HIV/AIDS, prevalence values for *C. belli* in stool samples ranging between 0.4% and 15% in a left-shifted distribution had been recorded previously [3,4,5,6,7,8,9,10,11,12]. Thereby, prevalence rates higher than 5% had been predominantly observed in symptomatic patients with advanced AIDS-associated immunosuppression as reported from Mali [10] as well as in HIV-patients in Central and South America [6,9] compared to lower prevalence rates in Sub-Saharan African [3,4,5,11] or South East Asian [7] HIV-patients.

The calculated test accuracy-adjusted *C. belli* prevalence of 3.2% for a population comprising predominantly Ghanaian HIV patients within different stages of the infection was well in the expected range and in line with previous reports [3,4,5,11]. The single positively tested stool sample from a subpopulation of 95 German soldiers after deployment in Central Africa, in contrast, confirms the expectation of low prevalence, because cystoisosporiasis is rare in subpopulations without specific preselection for immunosuppression [13]. Indeed, to the best of the authors’ knowledge, this is the first published case of a German soldier presenting with PCR-based evidence of *C. belli*, while a recent surveillance report on infectious diseases in German soldiers after tropical deployments failed to indicate any cystoisosporiasis cases [43]. As both applied PCRs detected a positive result, with Ct values of 35.31 in the 5.8S rRNA gene/ITS-2 sequence PCR and of 31.33 in the ITS-2 sequence PCR, respectively, a false positive result can be considered as unlikely. So, this case confirms the previous observation that individual cases of cystoisosporiasis in immunocompetent hosts have a high likelihood of going undetected in case of sole reliance on microscopic diagnosis [24], because the respective stool sample had been previously assessed by microscopy with a negative result for *C. belli* as reported elsewhere [43].

For both comparatively assessed real-time PCR assays, the applied latent class analysis suggested excellent specificity close to 100%. With focus on sensitivity, the 5.8S rRNA gene/ITS-2 sequence PCR scored slightly poorer than the ITS-2 sequence PCR, with calculated values of 92.8% and 100%, respectively. Although the low numbers of positive samples resulted in broad 95%-confidence intervals, the calculated sensitivity values obtained from the test comparison without a reference standard nearly perfectly matched previously reported values in comparison to microscopy of 93% for the 5.8S rRNA gene/ITS-2 sequence PCR assay [28] and of 100% for the ITS-2 sequence PCR assay [33], respectively. In contrast to a previous assessment [28], however, it could be shown that specificity of the 5.8S rRNA gene/ITS-2 sequence PCR is considerably better than the previously recorded 93% [28] and virtually 100% instead. This result is not surprising, as sensitivity restrictions of microscopy-based diagnosis of protozoa in human stool samples, even if performed by well-trained personnel from reference laboratories, have been reported [44,45], so it is likely that presumed false positive real-time PCR results from the previous assessment [28] had in fact indicated true positive stool samples which had gone undetected by microscopy due to low parasite loads.

Noteworthy, almost perfect agreement [39] between both assessed real-time PCR assays was recorded. Thereby, the discrepancies were simply due to 4 additional positive results of the ITS-2 sequence PCR, while all positive results of the 5.8S rRNA gene/ITS-2 sequence PCR could be confirmed by the competitor assay. So, if higher sensitivity is desired, the ITS-2 sequence PCR assay is preferrable, while the 5.8S rRNA gene/ITS-2 sequence PCR may be considered for confirmation testing.

Interestingly, the Ct values of the samples positive in the ITS-2 sequence PCR but negative in the 5.8S rRNA gene/ITS-2 sequence PCR were higher than the average value albeit lower than the maximum recorded Ct values in the ITS-2 sequence PCR. So, it remains questionable whether stochastic effects of target DNA quantities close to the limit-of detection alone may have accounted for the discrepant results. As differentiation between sensitivity and specificity problems may be challenging on an individual level, a positive result in the more sensitive ITS-2 sequence PCR alone may be considered to define a probable case of a *C. belli* infection. In case of an additional positive result in the slightly more specific 5.8S rRNA gene/ITS-2 sequence PCR, such a probable case could become a confirmed case. Considering the excellent specificity values of both PCR assays, however, a practical need for combined screening and confirmation testing for *C. belli* in stool is at least debatable and its usefulness will largely depend on the expected pretest probability as well as on the prevalence-depending predictive values in line with Bayes’ theorem [37].

The study has a number of limitations. First, microscopic results were not available for the most residual materials and thus could not be included as a reference standard. Accordingly, latent class analysis was chosen for a test comparison without a reference standard [37,38]. Second, although samples with a comparably high pretest probability were chosen for the assessments [40,41,42], cystoisosporiasis remains a rare disease. Accordingly, low absolute numbers of positive samples were recorded, leading to large 95%-confidence intervals in the LCA-assessments. Accordingly, considering the rare occurrence of the disease and the scarce availability of reference materials for respective test comparisons, a residual uncertainty regarding the reliability of the calculated test characteristics remains. Third, potential effects of nucleic acid extraction on the diagnostic sensitivity of the applied *C. belli*-specific real-time PCRs were not assessed, while such influences had been indicated by a previous report [36]. Although this report had suggested increased sensitivity after semi-automated EZ-1-based nucleic acid extraction (Qiagen, Hilden, Germany) compared to the column-based QIAamp DNA stool mini kit (Qiagen), we preferred the column-based nucleic acid extraction from the stool samples due to comparably lower sample inhibition rates, as reported elsewhere [46]. Indeed, the inhibition rates of the samples from the soldiers were in the expected range for column-based nucleic acid extraction with 2.1% (2/95) [46]. In contrast, the inhibition rate of the Ghanaian stool samples was moderately higher, with 3.4% (31/905); most likely a consequence of challenging sample storage and transport conditions associated with studies in the Sub-Saharan tropics as discussed elsewhere [37,47].

## 4. Materials and Methods

### 4.1. Study Population

A total of 1000 residual nucleic acid extractions from stool samples obtained from Ghanaian HIV patients (n = 905) (descriptions of the population in [40,41,42]) and German military returnees from tropical deployments in the Democratic Republic of the Congo and Gabon (n = 95) (description of the population in [43]) were included in the assessments in order to ensure an appropriate pretest probability. At the time of the test comparison, the samples were between 7 and 14 years of age. The residual DNA had been stored at −80 °C prior to the analyses. Microscopical results were not available. Patient-specific data such as age, sex, or medical history are not provided in line with the ethical clearance, allowing the use of fully anonymized residual materials for test comparisons only. This lack of information is an admitted violation of the STARD (Standards for Reporting Diagnostic Accuracy) criteria [48].

### 4.2. Nucleic Acid Extraction

Nucleic acids from the stool samples had been extracted using the QIAamp stool DNA mini kit (Qiagen, Hilden, Germany) as described by the manufacturer. After nucleic acid extraction, the eluates had been stored at −80 °C until the assessments.

### 4.3. Applied Real-Time PCR Assays

Two *C. belli*-specific in-house real-time PCR assays targeting a 213-base-pair fragment of the combined 5.8S rRNA gene/ITS-2 sequence [28] as well as an 89-base-pair fragment of the ITS-2 sequence alone [33] were applied in this study with all sample materials. The oligonucleotides are shown in Table 3.

Both assays were run on magnetic induction cyclers (MIC, Bio Molecular Systems Ltd., London, UK) applying the following protocol: Initial heating to 95 °C for 15 min followed by 45 cycles of denaturation at 95 °C for 15 s and annealing as well as amplification at 59 °C for 60 s with a subsequent final step of cooling to 40 °C for 20 s. The reaction mix consisted of HotStarTaq mastermix (Qiagen) and a final Mg^2+^ concentration of 5 mM for both assays. For the 5.8S rRNA gene/ITS-2 assay, primer concentrations were 0.3 pmol/µL and the probe concentration 0.2 pmol/µL; for the ITS-2 assay, primer concentrations were 0.06 pmol/µL and the probe concentration 0.2 pmol/µL. The assays were run in 20 µL volumes with 5 µL DNA eluate. Each run was accompanied by a positive control based on a plasmid (pEX-A128 vector backbone with the insert sequence a shown in the Table A1) and a negative control using PCR-grade water. An inhibition control PCR targeting Phocid herpes virus DNA as described previously [49] was run as well. The limits of detection (lod) of both assays were calculated applying a dilution series of the positive control plasmid and the software SciencePrimer.com (http://scienceprimer.com/copy-number-calculator-for-realtime-pcr, last accessed on 13 July 2021). Thereby, the calculated lod was <10 copies/µL for both assays.

### 4.4. Exclusion Criteria

Inhibited residual DNA samples as indicated by the applied inhibition control PCR [49] were excluded from further statistical assessments.

### 4.5. Statistics

Sensitivity and specificity of both assays as well as accuracy-adjusted *C. belli*-prevalence within the study population were identified applying latent class analysis (LCA) [37,38]. Fleiss’ kappa for the agreement between the assays was calculated and interpretated as described previously [39]. Further, the cycle threshold values of both assays were compared. The statistical analysis was performed using the software Stata/IC 15.1 for Mac 64-bit Intel (College Station, TX, USA).

### 4.6. Ethics

Ethical clearance for the comparative test evaluation with fully anonymized residual sample materials was provided by the medical association of Hamburg, Germany, (reference number: WF-011/19, obtained on 11 March 2019) without requirement for informed consent.

## 5. Conclusions

In conclusion, the performed test evaluation without a reference standard confirmed suitability of both assessed real-time PCR assays for the diagnosis of *C. belli* in human stool samples. If optimum sensitivity is desired, the ITS-2 sequence PCR assay should be applied. In contrast, the 5.8S rRNA gene/ITS-2 sequence PCR assay may be considered for confirmation testing due to excellent specificity if confirmation of a diagnostic detection of *C. belli* is intended.

## Figures and Tables

**Table 1:** Summary of the latent class analysis (LCA) results and Fleiss’ kappa recording the agreement between the assessments.

PCR	n	Positives (%)	Sensitivity (0.95 CI)	Specificity (0.95 CI)	Kappa (0.95 CI)
5.8S rRNA gene/ITS-2 sequence PCR	967	29 (3.00)	0.928 (0, 1)	1 (n.e.)	0.933 (0.868, 0.998)
ITS-2 sequence PCR	967	33 (3.41)	1 (0, 1)	0.998 (0,1)	
Prevalence (0.95 CI)	0.032 (0.010, 0.092)				

0.95 CI = 95% confidence interval. n = number. n.e. = not estimable.

**Table 2:** Cycle threshold (Ct) values as recorded for both *C. belli*-specific real-time PCR assays.

PCR	n	Mean (SD)	Median (Min, Max)
5.8S rRNA gene/ITS-2 sequence PCR	29	31.57 (4.14)	31.45 (23.98, 38.24)
ITS-2 sequence PCR	33	30.66 (4.27)	31.33 (23.62, 36.89)

0.95 CI = 95% confidence interval. n = number.

**Table 3:** Oligonucleotides as applied with the 213-base-pair fragment of the combined 5.8S rRNA gene/ITS-2 sequence [28] as well as with the 89-base-pair fragment of the ITS-2 sequence alone [33].

Forward Primer Name	Forward Primer Sequence	Reverse Primer Name	Reverse Primer Sequence	Probe Name	Probe Sequence
Real-time PCR targeting a combined 5.8S rRNA gene/ITS-2 sequence fragment of *C. belli*					
Cys Ib-213-F	5′-GGATATTCCCTGCAGCATGT-3′	Cys Ib-213-R	5′-CGGGACACAACTCAACACTG-3′	Cys Ib-213-P	5′-GTCACAGCGGCGTTTACGC-3′
Real-time PCR targeting the ITS-2 sequence of *C. belli*					
Cys Ib-40-F	5′-ATATTCCCTGCAGCATGTCTGTTT-3′	Cys Ib-129-R	5′-CCACACGCGTATTCCAGAGA-3′	Cys Ib-81-P	5′-CAAGTTCTGCTCACGCGCTTCTGG-3′

## Data Availability

All relevant data are presented in the article. Raw data can be provided on reasonable request.

## Appendix A

**Table A1:** Positive control plasmid insert used for the *C. belli*-specific real-time PCRs.

Positive Control Sequence Insert
5′-GGCGCTGTGGGGATATTCCCTGCAGCATGTCTGTTTCAGTGTCTCTGAAGTTTCAAGTTCTGCTCACGCGCTTCTGGGGGTGTCTCTGGAATACGCGTGTGGCAGTGTGACTGGATGTCTTGGGTGTTGAGAAACAAGCTACTTGTGCTTCTAGAAAGCCGAACGTCATCCGAAATAGTCACAGCGGCGTTTACGCGATCAAACAGTGTTGAGTTGTGTCCCGAACATCTTTG-3′
